# Proteomic Insights into Brain Development: Neurotoxic Effects of PBDE-99 in Mice

**Published:** 2006-02

**Authors:** Charles W. Schmidt

The amounts of polybrominated diphenyl ethers (PBDEs) found in human tissues, particularly breast milk, are rising steadily. PBDEs are flame retardants that reduce fire risks when added to electronic equipment and household products. Epidemiologic studies have yet to show conclusive evidence of PBDE toxicity in humans. However, studies in animals have shown these chemicals to produce effects such as liver damage, altered thyroid hormone levels, developmental changes, and neurotoxicity. In the present study, a Swedish team uses proteomic methods to investigate the early-stage neurotoxic effects of PBDE-99 on two parts of the developing mouse brain: the striatum and the hippocampus **[*EHP* 114:254–259]**. The findings help deconstruct the mysterious mechanisms that underlie PBDE-99 neurotoxicity.

The striatum and the hippocampus are both part of the cholinergic and monaminergic systems, which play roles in neurotransmitter functioning and other aspects of cognition. The authors sought specifically to uncover PBDE-induced cellular events leading from the neonatal brain growth spurt to permanent neurological problems in adult animals.

PBDE-99 is among the most common PBDE congeners found in environmental and human tissue samples. This pentabrominated compound impairs spontaneous behavior and habituation among adult mice exposed neonatally to moderate doses. These effects are progressive, worsening with age.

During the study, 10-day-old mice of both sexes were given a single dose of 12 milligrams PBDE-99 per kilogram body weight. After 24 hours, the mice were sacrificed, their brains dissected, and the striatum and hippocampus isolated for study. The team used 2D-DIGE to compare protein expression patterns between treatment and control groups. The analysis illuminated 40 proteins in the striatum and 56 proteins in the hippocampus whose expression was altered by PBDE-99 exposure.

From this initial grouping, nine striatal proteins and ten hippocampal proteins were selected for identification using MALDI-ToF-MS. Among the striatal proteins affected by PBDE-99 were several (including Gap-43/neuromodulin and stathmin) that participate in neurode-generation and neuroplasticity. The affected hippocampal proteins (including α-enolase, γ-enolase, Atp5b, and α-synuclein) tend to participate in metabolism and energy production. Many of these proteins are linked to protein kinase C, a signaling molecule whose role in development and function, as well as in learning and memory, has been intensively studied.

Based on these findings, the authors conclude they have identified potential protein biomarkers that reflect the immediate consequences of early-stage PBDE-99 toxicity, in addition to the processes that drive its neurological effects in older animals. The researchers suggest that protein kinase C signaling is a target of PBDE-99 toxicity in the developing mouse brain. Moreover, the authors propose that neonatal cell stress induced by PBDE-99 exposure, in addition to related neurodegenerative processes and aberrant neuroplasticity, may contribute to the latter-stage behavioral effects observed in adult mice. The responses within the striatum and hippocampus differ, however, reflecting the underlying heterogeneity between different brain parts and cell populations.

## Figures and Tables

**Figure:**
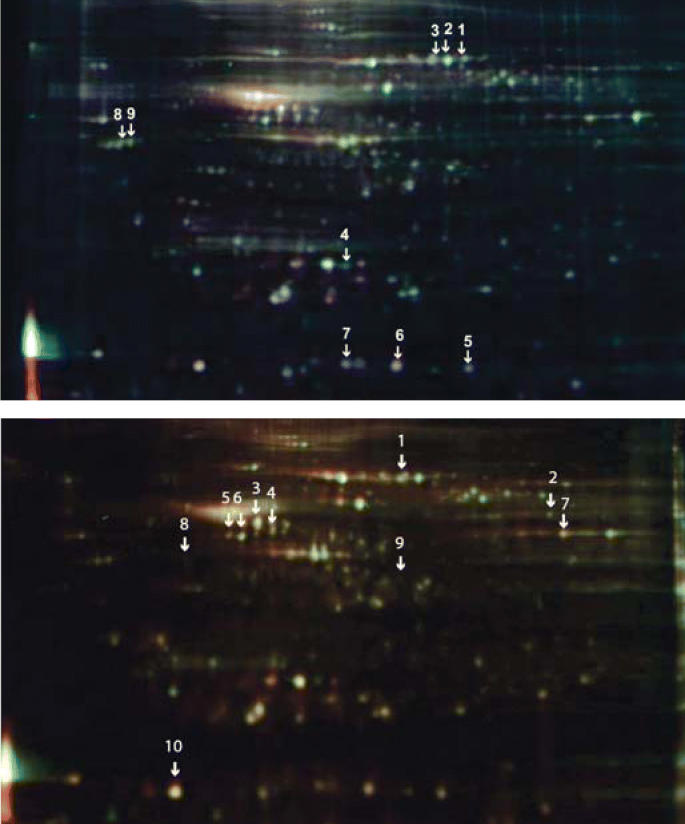
Lights in the darkness. 2D-DIGE reveals nine striatal proteins (top) and ten hippocampal proteins (bottom) that may serve as biomarkers of early-stage PBDE-99 toxicity.

